# Rotational hyperspectral scanner and related image reconstruction algorithm

**DOI:** 10.1038/s41598-021-82819-8

**Published:** 2021-02-08

**Authors:** Longqiang Luo, Shuo Li, Xinli Yao, Sailing He

**Affiliations:** 1 College of Optical Science and Engineering, Zhejiang University, 866 Yuhangtang Rd, Hangzhou, 310058 China; 2 Ningbo Research Institute, Zhejiang University, Ningbo, 315100 China; 3 Department of Electromagnetic Engineering, School of Electrical Engineering, KTH Royal Institute of Technology, 100 44 Stockholm, Sweden

**Keywords:** Imaging and sensing, Optical spectroscopy

## Abstract

We design and implement a compact and lightweight hyperspectral scanner. Based on this, a novel rotational hyperspectral scanner was demonstrated. Different from translational scanning, rotational scanning is a moveless and stable scanning method. We also designed a relevant image algorithm to reconstruct the image from an angular recorded hyperspectral data cube. The algorithm works well even with uncertain radial and tangential offset, which is caused by mechanical misalignment. The system shown a spectral resolution of 5 nm after calibration. Finally, spatial accuracy and spectral precision were discussed, based on some additional experiments.

## Introduction

Hyperspectral imaging spectrometer is a kind of scientific instrument, capable of acquiring spectral and spatial information synchronously. More than the output photo from a common color camera, hyperspectral image contains not only the intensity of RGB colors but also spectral data, which plays an important role in many industrial fields^1^ and research areas^2^. Since hyperspectral techniques were widely applied in food quality assessment^3–5^, environment monitoring^6,7^, agriculture^8,9^, and medical science^10–14^, new methods to acquire hyperspectral images are desirable.

Conventional hyperspectral imaging spectrometer acquires spectral data cube either by spectral scan or by spatial scan. In order to achieve spectral scan, researchers insert an acousto-optical tunable filter (AOTF) between the imaging lens and camera CMOS, so that they can get an image of an object in a certain spectral band^15–18^. The images acquired in each band is very clear, and post processing to obtain hyperspectral data cube is quite easy, simply stacking up images of different spectral bands. However, the transmission of the filter is low, and consequently the image integration time would be longer. Furthermore, the high price of AOTF limits the industrial applications.

Different from the AOTF method mentioned above, a spatial scan spectrometer generally records the spectra of a line in the object plane once a time and then stitching them together^19,20^. For example, in a push-broom hyperspectral spectrometer^21^, there is a linear slit to realize the spatial filtering, which is perpendicular to the sweep direction. As it mechanically sweeps through the entire object plane, a hyperspectral image is obtained. Hence, it usually needs a stable and precise translation stage and enough space to operate the sweep. The spatial resolution depends on the width of slit, which on the contrary must be wide enough to transit enough light to the camera. For some applications, this type of spectrometer is mounted on an airplane or vehicle to obtain the hyperspectral image of some ground surface or buildings^22^. However, due to the vibration during the translational spatial sweep, the output hyperspectral image jitters and is usually blurry, which is not as clear as the output from the spectral scan.

Apart from scientific usage, there is also a growing trend towards portable or hand-hold hyperspectral imaging spectrometer^23–25^, which will widely expand its application. Contrary to scientific hyperspectral imaging equipment, it must be light in weight and compact in structure. Some research groups have even already adopted hyperspectral imaging spectrometers on an unmanned aerial vehicle to obtain image with spectral information^26^. Recently, a semi-rotational scanning hyperspectral imaging spectrometer was reported^27^, and its optical dispersive elements worked in a rotational manner while the camera kept still. However, the output image was very much distorted, partly due to the relative movement between the camera and the rotational platform, and partly due to the lack of a proper image reconstruction algorithm. In the present paper, to obtain a better hyperspectral image we propose a more stable hyperspectral scanner with the camera rotated together with the rotational platform, as well as a proper image assembling algorithm.Considering the growing demand for industrial and consumer use, in the present paper we design and implement a compact hyperspectral scanner (150 mm in length and about 130 g in weight). Furthermore, we propose a rotational scanning method to capture and assemble hyperspectral images. In this way, translational mount is substituted by a rotational mount, where the scanner is placed. The scanner rotates together with the rotational stage along the optical axis while collecting images continuously.

Compared with spectral scanner, spatial scanner usually can reach a higher spectral resolution, which is based on the ability of the dispersion element employed in the device. Furthermore, the band width and transmission of a tunable filter usually vary with the wavelength. Thus our system based on a spatial scanner would achieve a higher spatial resolution. More importantly, a spatial scanner is more flexible. For example, our system is compatible with an image lens of any focal length, and suitable for objects of any distance. As long as we can carefully adjust the working distance, a clear image can be formed on the slit plane.

Furthermore, compared with a translational motion, our rotational movements is more stable, and can operate in a quasi–static manner. Furthermore, in order to assemble the image acquired in a rotary manner, we establish the corresponding geometric graphics algorithm. As demonstrated in the experiments, the assembled hyperspectral image is clear, and the spectra data is accurate. More importantly, the optical aberration of the output image is symmetry and isotropic, which is determined by the distortion of the image lens. This is quite different from the spatially-stacked image output, whose optical aberration in the pile-up direction is determined manually.

In this paper, we build up a kind of rotational hyperspectral scanner, and demonstrate its performance and output. It is shown that high performance can be achieved with a compact optical design and robust geometric graphics algorithm. It should be noted that this is the first time that a rotational scanning image reconstruction algorithm is demonstrated in practice, and it will provide fundamental help for other rotational scanning methods.

## Experiment details

### Optical design of rotational hyperspectral scanner

**Figure 1:**
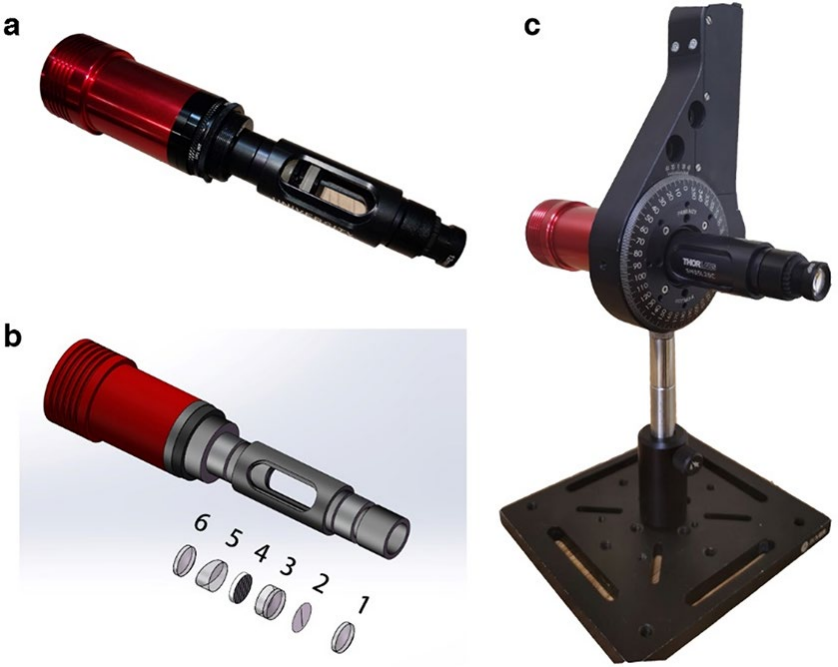
(**a**) The photo of our rotational hyperspectral scanner; (**b**) schematic diagram and optical elements (1. imaging lens; 2. line slit; 3. collimating lens; 4. grating; 5. prism; 6. imaging lens); (**c**) Installed on a rotational mount.

A photo of our portable hyperspectral scanner is shown in Fig. 1a. It consists of a customized imaging lens set and a grating-prism dispersion component, and its schematic illustration is presented in Fig. 1b. All optical components were placed inside a 1/2-in. tube, connected to the camera by a threading adapter. As described in Fig. 1b, the imaging lens (1; f = 12 mm) gives an image of the object onto the slit plane (2; d = 50 μm) near its focal spot. The image within the slit range goes through the slit and is collimated by a doublet lens (3; f = 30 mm) located 30 mm behind the slit plane, and the rest of light outside the slit range is filtered away spatially by the slit plane. This doublet lens was designed to be achromatic so that rays of different wavelengths are refracted at the same angle. Hence, the beam travels paralleled (without being dispersed) to the optical axis behind the doublet lens. After that, a grating (4; 300 lines/mm) is used to disperse the light according to the wavelength. For the beam with a wavelength of 500 nm, the first order diffraction angle is about 9.6°. Hence, a prism (5; 10°) is placed closely behind the grating, to rectify the first order diffraction beam onto the image plane. Finally, in order to focus the dispersed beam clearly onto the camera (ASI120MM-MINI, ZWO, China), we placed another imaging lens (6; f = 25 mm) in front of the camera. To reduce the size of the whole system, we make the distance between the lens and prism as short as possible. However, the distance between the imaging lens and camera must be equal to the focus length, which needs to be adjusted finely. The size of the gray CMOS (with a 12-bit digital output at each pixel) inside the camera is 1/3", and the output raw image is 960 pixels in height and 1280 pixels in width. Thus, the output hyperspectral image would have a high dynamic range in intensity.

The system is light-weight and compact, so that it can be inserted into a rotational mount (PRM1/MZ8, Thorlabs, USA), as we can see from Fig. 1c. The minimal achievable incremental motion of the rotational mount is 25 arcseconds. Meanwhile, the image of the slit is 12 pixels in width and 960 pixels in height, as we can see in the calibration process. We compute the arc corresponding to the width of the slit at the two ends, relative to the center of the slit. The angle of the arc at one end of the slit is about 2°. In order to get a complete image (even at the outmost edge), the rotational angle at each step of frame should be less than the angel of the arc. In the experiment, we usually set the motion increment to 1°. Hence the spatial resolution is not restricted by the motion step, but determined by the slit width. Different from push-broom scanning system, our rotational hyperspectral scanner rotates along the primary optical axis when working. The whole system, from the very front image lens to the camera, rotates one cycle to acquire an entire image of the object. During the rotation process, the direction of the slit varied, and the spectral images were captured synchronously.

### Spatial rotational image reconstruction algorithm

**Figure 2:**
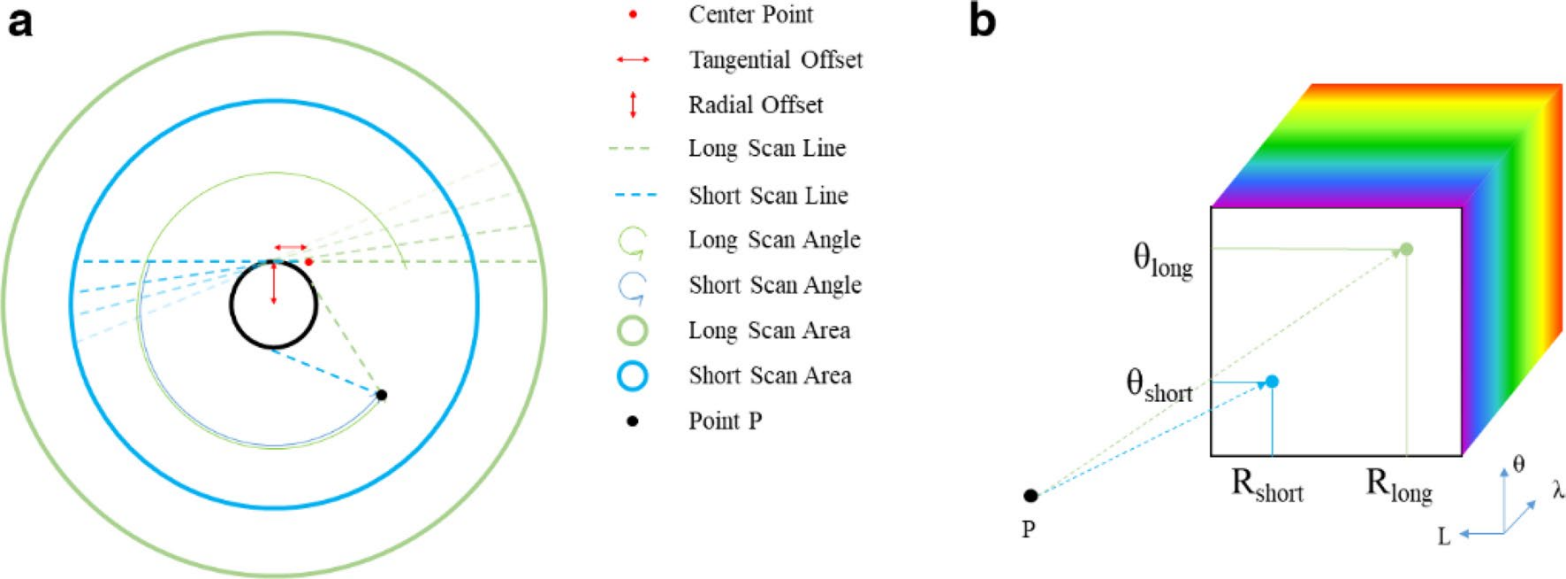
(**a**) Scanning with the rotational image line; (**b**) map to hyperspectral data cube.

Same as all other spatially scanning hyperspectral imaging spectrometers, each spectral image obtained corresponds to a line in the object plane. However, our rotational one has a unique arrange order. According to the mechanical rotation mentioned above, such a scan line revolved around a certain center point when the system works. As illustrated in Fig. 2a, the dashed line refers to the scan line, and it faded counterclockwise to indicate the process of scan. The scan line rotates 360° to complete one scan process, and thus we record the data cube with three dimensions of L, θ and λ, as shown in Fig. 2b. Furthermore, due to limited assembly accuracy, the center of the slit was not on the mechanical rotation axis. In terms of image formation, that means there are radial and tangential offsets between the scan line and the rotation center. Consequently, there exists a blind circle in the middle of the image.

Now, it is important to design an image algorithm to reconstruct the real image of the object from the data cube and estimate the two offset distances. As illustrated by vertical and horizontal double-headed arrows in Fig. 2a, the radial and tangential offsets are denoted by ${R}_{offset}$ and ${\mathrm{L}}_{offset}$, respectively. Assume the length of the image line equals to $2\mathrm{L}$, then the length of the long and short scan lines are $\mathrm{L}+{\mathrm{L}}_{offset}$ and $\mathrm{L}-{\mathrm{L}}_{offset}$, which is indicated by the green and blue dashed lines, respectively. For each point P $({\mathrm{R}}_{p}, {\theta }_{p}\in \left[\mathrm{0,2}\pi \right))$ within the range ${\mathrm{R}}_{p}^{2}<{R}_{offset}^{2}+{(L+{L}_{offset})}^{2}$, it can be swept across by the longer scan line. Thus, we can calculate the rotation angle ${\uptheta }_{long}$ and its relative location ${\mathrm{R}}_{long}$, when point P is scanned by the longer scan line. ${\mathrm{R}}_{long}$ equals to the tangent distance of point P and blind circle, and ${\uptheta }_{long}$ is the angle between the current scan line and the initial scan line, assuming that the scan initiates from the horizontal dashed line and rotates counterclockwise (as positive θ)

1 $${\mathrm{R}}_{long}=\sqrt{{R}_{p}^{2}-{R}_{offset}^{2}}$$

2 $${\theta }_{long}={\theta }_{p}-{\mathrm{sin}}^{-1}({R}_{offset}/{R}_{p})$$

According to ${\uptheta }_{long}$ and ${\mathrm{R}}_{long}$, we can map any pixel in the image plane to a certain point in L-θ plane of the data cube, as shown in Fig. 2b. In other words, we can establish the relation between the L-θ coordinates of the hyperspectral data cube to the polar coordinates of the image, with consideration of the radial and tangential offset. Furthermore, a linear interpolation algorithm was adapted to solve the problem of mapping discrete image pixels to discrete raw data cube.

### Parameter optimization

**Figure 3:**
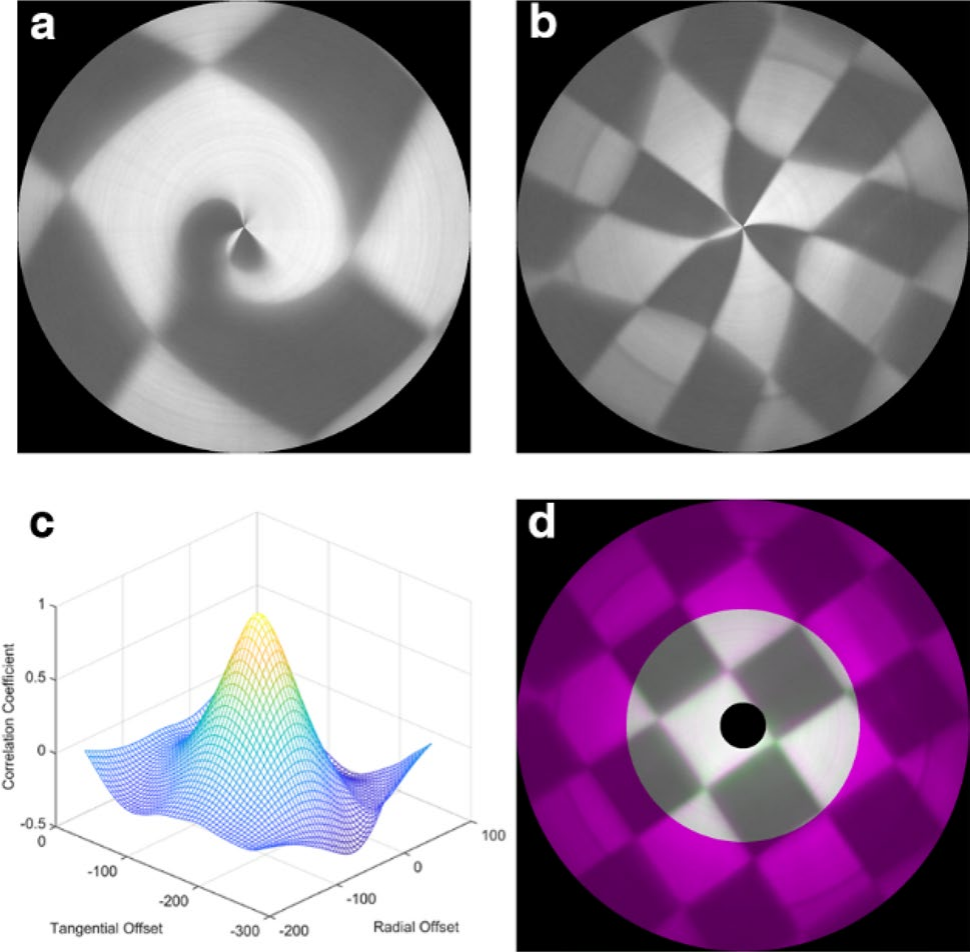
(**a**) Reconstructed image by short scan line; (**b**) reconstructed image by long scan line; (**c**) correlation coefficient chart; (**d**) optimum reconstructed image.

We can reconstruct the image from the data cube with the method mentioned above. However, without setting optimal radial and tangential offsets, the output image is usually distorted and deformed, as shown in Fig. 3a and b. When we take a photo of a checker board, the output image shrinks or expands, and a straight line bent.

Notice that the image inside the short scan area (i.e., P $({\mathrm{R}}_{p}, {\theta }_{p})$ within the range ${\mathrm{R}}_{p}^{2}<{R}_{offset}^{2}+{(L-{L}_{offset})}^{2})$ in Fig. 2a was swept by both the long scan line and the short scan line. Thus, we can also calculate its location ${\mathrm{R}}_{short}$ and rotation angle ${\uptheta }_{short}$ relative to the short scan line at the same time, as shown in Fig. 2b by the blue dashed line. Our optimization strategy is to analyze the similarity of the two images reconstructed by the long scan line and the short scan line. Thus, we calculated the correlation coefficient of the two images. A higher correlation coefficient indicates higher similarity. If the two images were very similar to each other, it means that both of them were similar to the real object with little distortion.

By varying ${R}_{offset}$ and ${\mathrm{L}}_{offset}$, and computing their correlation coefficient, we draw a grid chart of the correlation coefficient in Fig. 3c. We determine ${R}_{offset}$ and ${\mathrm{L}}_{offset}$ from the location of the peak of the correlation coefficient, and reconstruct the image shown in Fig. 3d. It is a composite RGB image showing two images overlaid in different color bands. Gray regions in the composite image show where the two images have the same intensities. Magenta and green regions show where the intensities are different. The image reconstructed by the long scan line is larger than the one by the short scan line, and thus the outer part (in magenta color) of the image is only from the larger image reconstructed by the long scan line. We should pay more attention to the inner part where the two images are overlapped. The well overlapped result in Fig. 3d shows that the intensity and shape of these two images are well matched.

Compared with conventional image assembling methods, our system actually scans the object twice. Thus we get two images pattern for self-correlation. By computing the difference of these two images with varied parameters R_offset_ and L_offset_, we can get a pair of optimized parameters and obtain an optimized self-correlated hyperspectral image. Hence the image output is better in stabilization and blur reduction. On the other hand, the rotational step is quite small, so that the relative movement between two scanning lines may be within one pixel. We average these points inside one pixel, so that the output image becomes smooth and clear. More importantly, the output image has a fixed magnification in two directions determined by the image system, while the magnification of translational assembled images is determined manually in the sweep direction.

## Result and discussion

### Calibration

**Figure 4:**
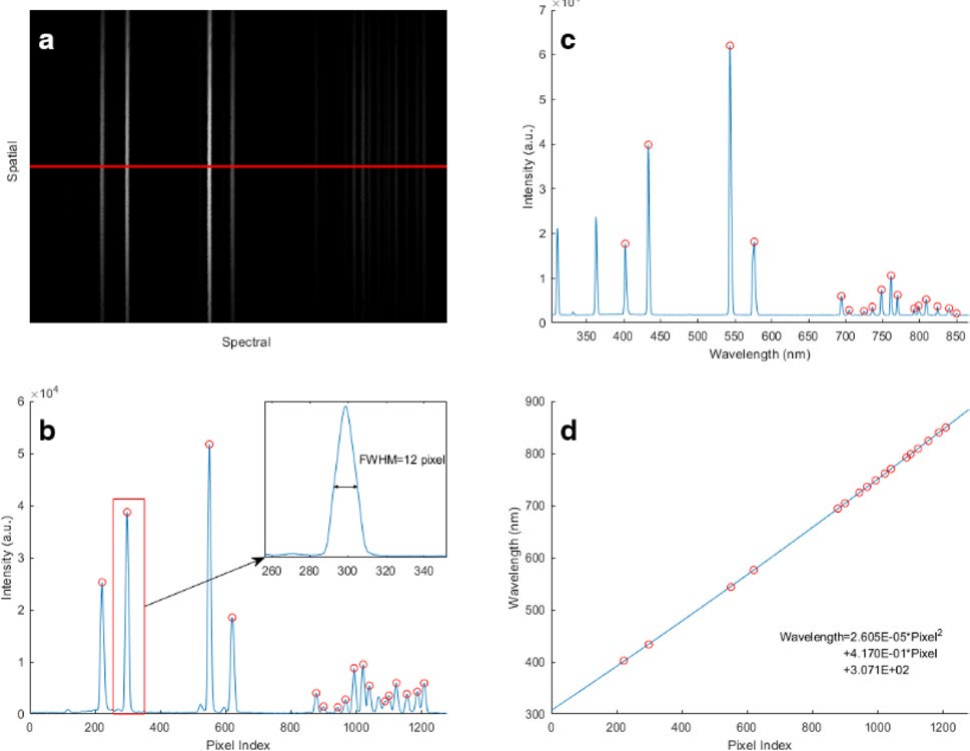
Calibration with an Hg–Ar lamp. (**a**) raw image; (**b**) intensity distribution on pixels; (**c**) spectrum measured by our spectrometer; (**d**) polynomial fitting result.

For all hyperspectral imaging spectrometer, it is imperative to calibrate before practical use. Thus we use a collimated beam of light from Hg-Ar lamp directly incident onto the slit, and record the image of CMOS output. The raw image is shown in Fig. 4a, and the vertical sharp lines correspond to the spectral emission lines of the Hg–Ar lamp.

We plot the intensity distribution over the pixel of the image center, which is marked with a red line in Fig. 4a. As we can see from the results in Fig. 4b, there are 17 peaks distributing over 1280 pixels. On the other hand, we measure the spectrum of the Hg–Ar lamp with a spectrometer (USB 2000+, Ocean Optics, USA), and plot it on Fig. 4c.

We chose (with circled tips) a group of significant peaks in Fig. 4b,c, and use them in the following calibration process. We fitting the relationship between the wavelength and pixel index value with a two-order polynomial, and present the result in Fig. 4d. The fitting equation is $\mathrm{y}=2.605\times {10}^{-5}\times {x}^{2}+4.170\times {10}^{-1}\times x+3.071\times {10}^{2}$, where y is the wavelength and x is the pixel index. As we can see from the plot, all the circled point is very close to the fitting line, which means the fitting is perfect. Furthermore, the first order coefficient is much higher than the second order coefficient, and it is almost linear fitting, which confirms that the optics was installed properly and precisely. The peak indicated by a red rectangle in Fig. 4b, shows an FWHM of 12 pixels, and thus we can calculate that the spectral resolution is 5 nm. The spectral resolution depends heavily on the width of the slit. Nevertheless, a thin slit will also lead to an extended exposure time. Considering the dynamic range of the camera, we choose a slit of 50um to achieve the spectral resolution of 5 nm.

Furthermore, the spectral resolution is also determined by the dispersion ability of the grating. However, enlarging the dispersion ability by adapting a grating with a larger number of line pairs will reduce the spectral measuring range, since the length of CMOS is definite. The spectral measuring range is from 307 nm to 883, which just covers the visible range. It is well matched with the response range of the camera, satisfying the requirement for common scenario.

### Image reconstruction

To verify our image reconstruction algorithm, we took a photo of a building, 78 m away from the imaging spectrometer in our campus. Since the sunshine was strong, we set the exposure time to 10 ms. During one rotational cycle, our camera record 361 images. The size of the hyperspectral data is enough for spatial resolution, meanwhile efficient for computing.

**Figure 5:**
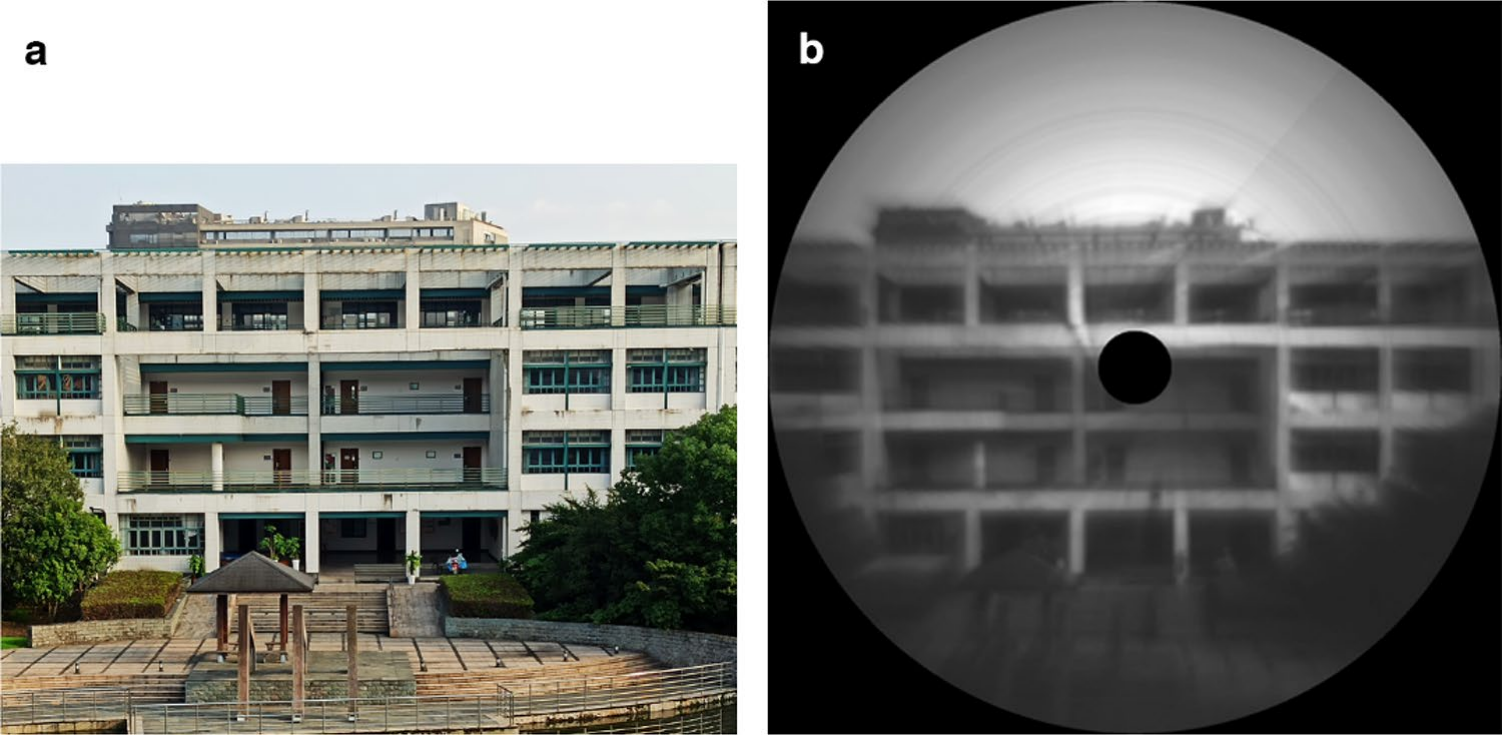
(**a**) Photo of a building; (**b**) image reconstructed by our rotational hyperspectral scanner.

Figure 5a is the photo of the building, while Fig. 5b is the reconstructed hyperspectral image. As we can see from the gray scale image, except that the inevitable missing piece in the center of the image, no noticeable distortion or deformation was observed. The resolution of the image is mainly limited by the width of the slit. However, a narrower slit reduces the incident light and much longer exposure time is needed. Thus we choose a 50-um-width slit, as a compromise of exposure time and spatial resolution. Hence, the angular resolution is 4.17 × 10–3 rad, which is calculated through dividing the slit width (50 um) by the focus length (12 mm) of the imaging lens. Thus we can estimate that the spatial resolution for a target at 78 m away is about 32.5 cm. However, due to the rotary manner in image stitching process, the pixel distribution around the center of the image is much denser than those near the edge. Consequently, we can find the sharpness of the output image decreases with the radius. In addition, there are some concentric circle fringes in Fig. 5b. That is due to the burrs of the metal slit, which result in different amounts of incident light along the slit. In general, it is impressive to achieve such a spatial resolution without distortion by a rotational hyperspectral scanner.

### Color restoration

**Figure 6:**
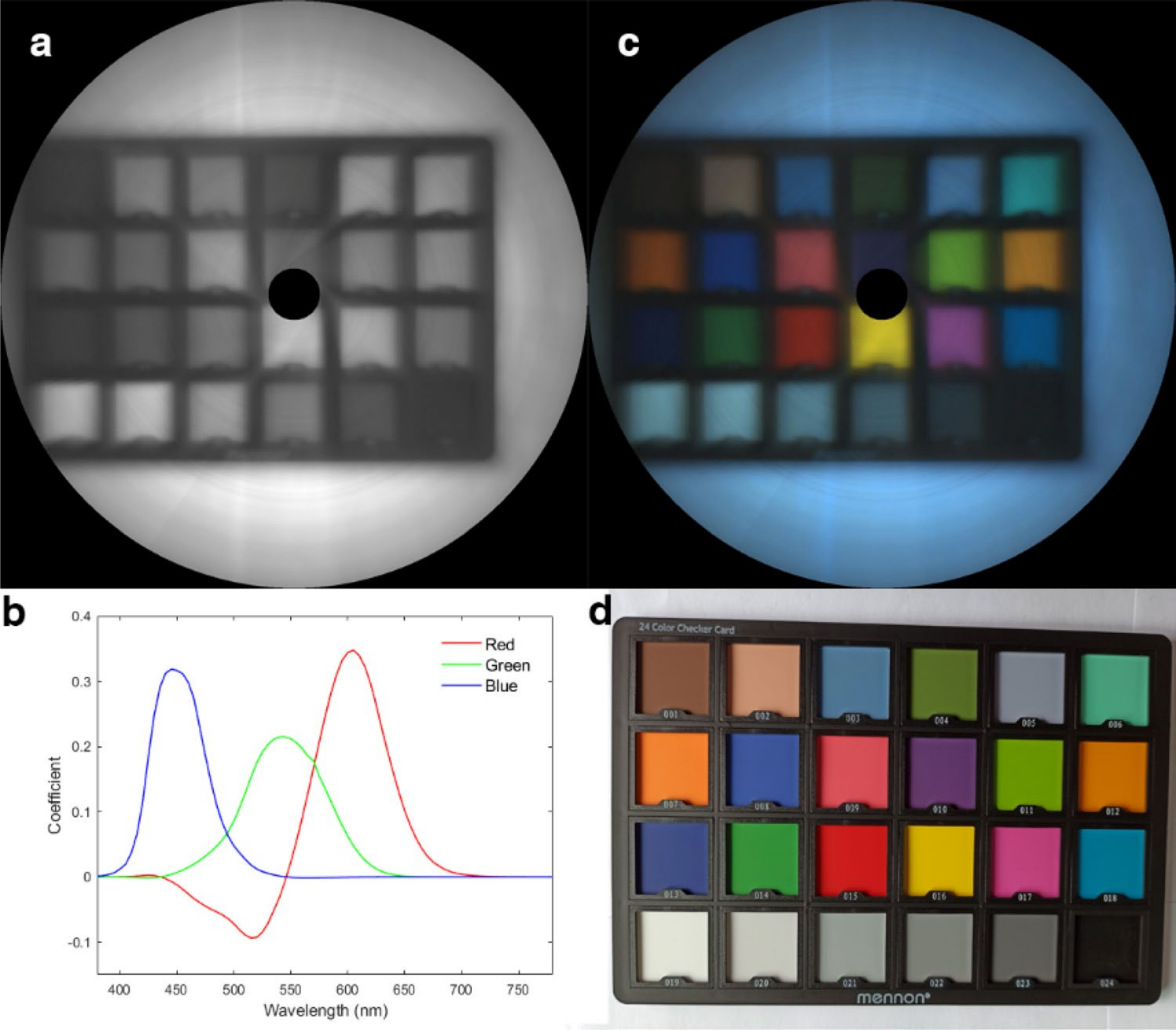
(**a**) Full spectral image; (**b**) CIE-1931 RGB stimulation coefficient; (**c**) color restoration; (**d**) color checker.

Besides the spatial accuracy of our rotational hyperspectral scanner, we also need to verify its color accuracy. Therefore, we ran an experiment with a color checker. We put the color checker on a white paper and use our rotational hyperspectral scanner to capture its reflection, whose full spectral image is shown in Fig. 6a.

Since lights of different wavelengths have different visual stimulation, we usually perform the color restoration referred as the CIE-1931 RGB coefficient, which is plot in Fig. 6b. It describes the color stimulation coefficient in visible wavelengths. We put the image after color restoration in Fig. 6c, and the photo of the color checker is also given in Fig. 6d for comparison.

We can distinguish every color on the checker board clearly, even the gray scale difference. It is well demonstrated that our system has a proper intensity response, even when the spectral intensity have not been calibrated. This is credited to the flat response curve of the CMOS. The hyperspectral data cube is directly converted from the raw output from a gray camera of 12-bit digital output, and the dynamic range is much higher than a color camera. Although the cold tone over the entire restored image may result in color inconsistency between Fig. 6c and Fig. 6d, which is probably due to the lack of proper white balance. Overall, the color restoration is successful, which verifies a high spectral performance of our rotational hyperspectral scanner.

### Application

**Figure 7:**
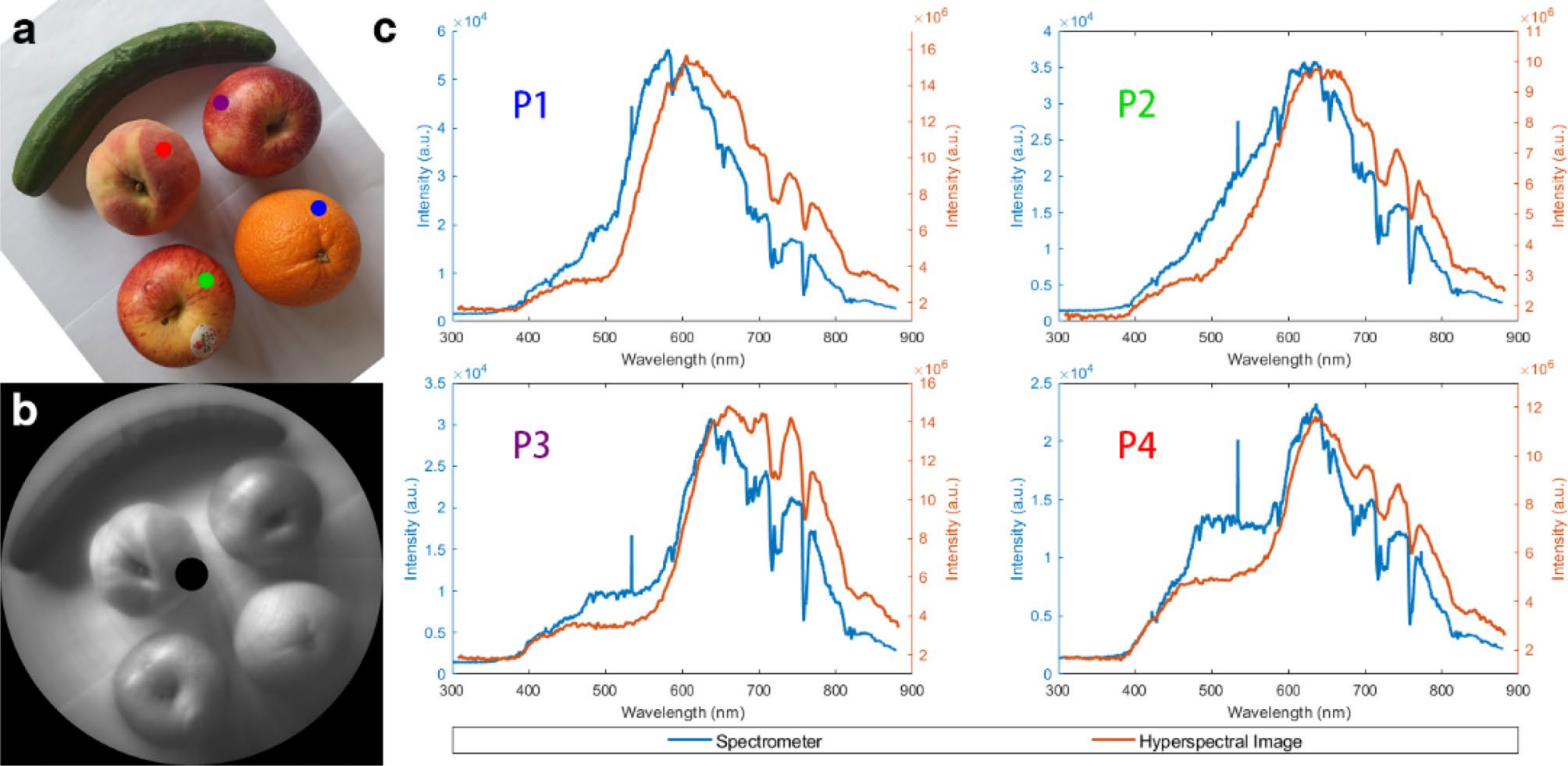
(**a**) Photo of fruits; (**b**) hyperspectral image; (**c**) spectra plot.

Furthermore, we took a photo of two apples, a banana, a pear and a cucumber on one plate. Figure 7a shows the photo of the fruits, and Fig. 7b shows the image result from our rotational hyperspectral scanner. Meanwhile, we also use a spectrometer to measure the spectra of the fruits to make comparisons, at four specific test points marked with dots in Fig. 7a. In Fig. 7c, we plot the spectra obtained from the hyperspectral image data cube with blue lines, while the spectra captured by the spectrometer with orange lines. As we can see, the shapes of those pairs of spectral curves are quite similar, and some unique dips at 710 nm and 760 nm were observed, which are due to the illumination source. Yet, the results from our hyperspectral image are slightly higher than those from the spectrometer in longer wavelength band over 700 nm. We believe this is due to the higher sensitivity of the CMOS in our rotational hyperspectral scanner. Comparing the four plots in Fig. 7c, it is obvious that our hyperspectral images have potential to distinguish more clearly fruits of similar color, according to their spectral data.

**Figure 8:**
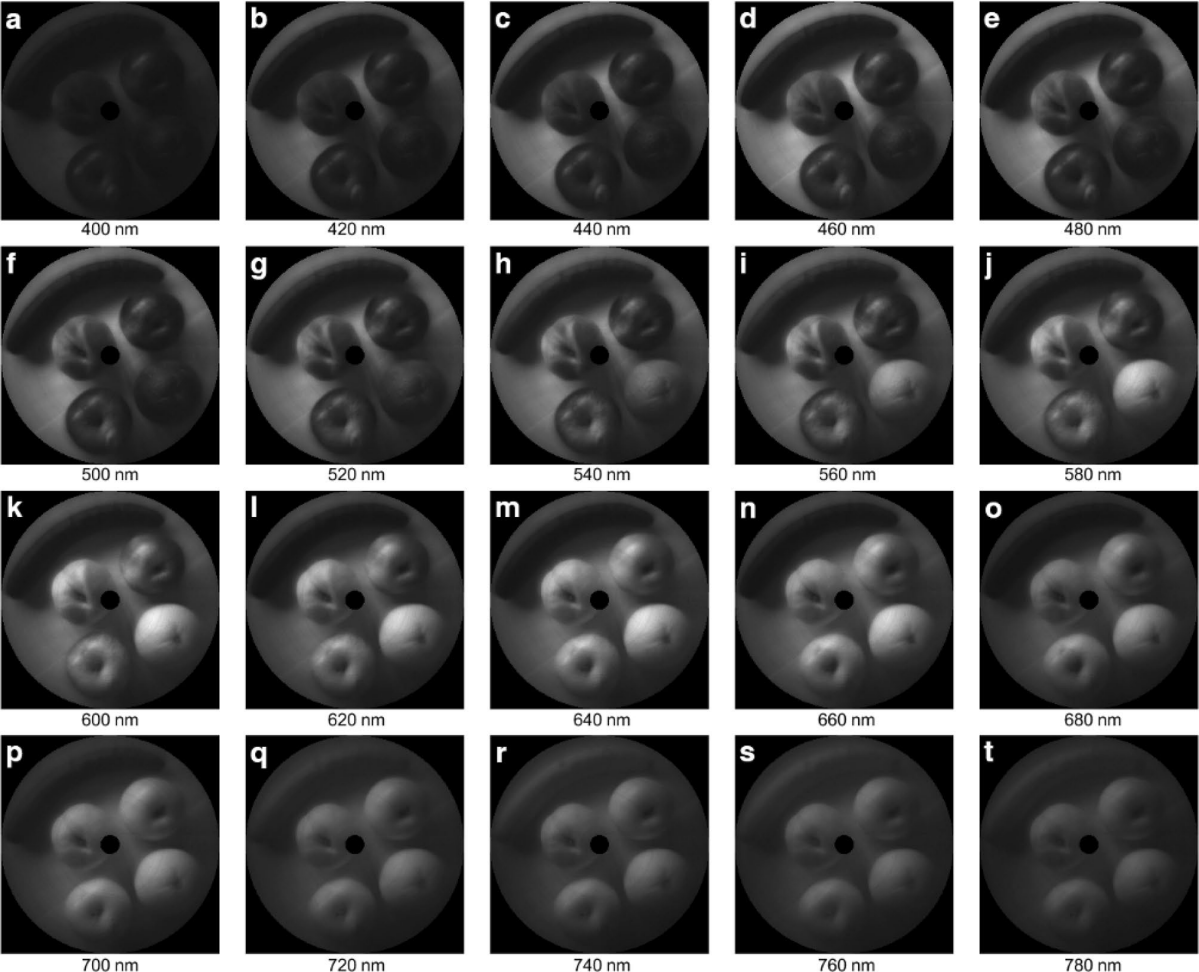
spectral image at different wavelength bands.

We also pick a series of wavelength bands from the data cube, and show the image at these bands, to verify its performance further. Figure 8 shows the fruit image at different wavelength bands. Each band has a width of 20 nm and an interval of 20 nm from the neighboring band, and all these bands cover the entire visible light range, from 400 to 780 nm.

Consistent with the spectra of the fruits, the orange shows its maximal brightness at 600 nm, and the cucumber at 540 nm and 720 nm. At 580 nm band, we can sharply distinguish the red and yellow patterns on the apple. Overall, the fruit reflect most of the illumination light around 600 nm.

The output image of different wavelength band is consistent with the color of appearance. Furthermore, the image of full spectrum looks the same as the color image. This further proves that our rotational scanner can acquire the hyperspectral image with high spatial and spectral accuracy.

## Conclusion

In this work, we have developed a rotational hyperspectral scanner, which is very compact and light weight, and adapted on a rotational mount. The imaging spectrometer scans the object of interest rotationally through the rotating mount. The slit gated the image of the object with different angle, and the spectral data are recorded, as well as the spatial information of the scan line. This way we obtain the spatial and spectral information of the object.

As we all know, rotational movement is more stable and less complex than conventional translational movement. 3D shape reconstruction is another hot topic recently^28–30^. Instead of translational scanning, our rotational hyperspectral scanner works in a gaze (staring) state (the central location is fixed in a 3D coordinate system) without any filter. Combining with another camera, such a hyperspectral imager working in a gaze state can be utilized for 3D shape reconstruction with hyperspectral information.

More importantly, we have designed an algorithm to reconstruct the image from the recorded data cube. To solve the misalignment problem between the rotation axis and center of the slit, we have preset radial and tangential offset parameters and compared the similarity of the images reconstructed through the long scan line and short scan line, by calculating their correlation coefficient. Once the optimal radial and tangential offsets are determined, an accurate image can be reconstructed.

We have also carried out some experiment to verify the performance. In order to determine the wavelength precisely, we have calibrated the wavelength with pixel index by two-order polynomial fitting. Furthermore, we have verified the image reconstruction ability and color restoration ability. As an example, we have also acquire some hyperspectral images of fruits.

Our rotational hyperspectral scanner is innovative in both mechanical structure and image algorithms. Its affordability and robustness will open a wide application for our rotational hyperspectral scanner.
